# Study of the use of a personalized peripheral sealing device on surgical face masks in high-risk situations against COVID-19

**DOI:** 10.1371/journal.pone.0253382

**Published:** 2021-08-06

**Authors:** Pere Riutord-Sbert, Thais Cristina Pereira, Joan Ernest de Pedro-Gómez, Diego González-Carrasco, Angel Arturo López-Gónzalez, Pål Barkvoll

**Affiliations:** 1 ADEMA School of Dentistry, University of the Balearic Islands, Palma, Balearic Islands, Spain; 2 Department of Nursing and Physiotherapy, University of the Balearic Islands, Palma, Balearic Islands, Spain; 3 Occupational Risk Prevention Service, Balearic Islands Health Service, Palma, Balearic Islands, Spain; 4 Department of Oral Surgery and Oral Medicine, Faculty of Dentistry, University of Oslo, Oslo, Norway; China University of Mining and Technology, CHINA

## Abstract

A significant number of health care professionals subjected to high-risk situations have been infected by Covid-19 due to the lack of adequate protection equipment or the deficient safety margins that these present. The aim of this study was to investigate whether the use of a personal peripheral sealing device (PSD) on surgical face masks (SM) allows them to achieve double mask properties, by providing two-way protection to professionals or users. The proposed device is a thermoplastic resin ring composed of a reusable and biodegradable polylactic acid (PLA) designed to be used in a healthcare setting. Since it is a thermoplastic device, it can be molded and adapted to each individual, becoming personalized and ensuring a correct adjustment to the user’s face. First, a qualitative fit test was performed using a saccharin solution (SS) to evaluate respiratory protective equipment in recruited professionals exposed to high-risk situations of infection by Covid-19. Individuals were divided into an intervention group, who used SM with the PSD, and a control group, who used SM without the PSD. In addition, a quantitative inward air leakage fit test was performed using a 2% sodium chloride (NaCl) aerosol in a sealed cabinet with probes sensitive to this substance, in order to validate the SM with the PSD as a Face Filtering Mask (FFP). Only 5% of the individuals who performed the qualitative fit test with the PSD perceived the sweet taste of the SS, while 100% of the individuals who performed the test without the PSD sensed it (*p* = 0.0001). In the quantitative fit test, the percentage of air leakage of 2% NaCl aerosol into the SM with the PSD was 6.5%, achieving the same range of air leakage as a FFP mask. Thus, the use of a personalized PLA thermoplastic PSD, together with an inexpensive and widely available SM, could have a significant impact in terms of preventive safety by providing bi-directional protection to its user.

## Introduction

The pandemic caused by the Severe Acute Respiratory Syndrome Coronavirus 2 (SARS Cov-2) or Coronavirus Disease 2019 (Covid-19) has had a direct impact on the world health system, causing an alarmingly high demand for Personal Protective Equipment (PPE) [1–3]. This increased demand has caused a global depletion of face mask reserves [4, 5]. Health professionals are considered a highly exposed and vulnerable group to Covid-19 [6], due to the nature of their work, and are classified by the Occupational Safety and Health Administration (OSHA) of the United States of America (USA) as having a “very high risk” of infection [7]. Thus, new protection requirements have been implemented with the mandatory use of filtering face masks (Filtering Face Piece 2 (FFP2) or Filtering Face Piece 3 (FFP3)), replacing the surgical face masks (type II and IIR) previously used by these professionals [8].

Among the most common forms of transmission of Covid-19, the most critical for health professionals is through direct actions where the patient’s oropharyngeal secretions or exudates are present, whereby the quantitative presence of Covid-19 is proportional to the virus load from infected people. These secretions are transmitted to the environment by Flügge drops expelled with expiration, speech, or cough. There is strong scientific evidence that Covid-19 can also be transmitted via other means [9], such as the aerosols generated by rotating instruments cooled with water in medical interventions of the oral cavity and upper respiratory tract [10, 11].

In this sense, the surgical face masks commonly used by health professionals before the current pandemic do not guarantee adequate protection against Covid-19, mainly due to their poor marginal fit or peripheral sealing. Thus, FFP2 and FFP3 masks are recommended by health authorities, because they provide an acceptable marginal sealing [12]. However, this is only achieved with optimal fitting efficiency, which has been found in less than 20% of professionals or users [13] due to the fact that they are preformed, standardized, and not individualized.

To date, no conclusive studies have been published that compare the effectiveness of surgical face masks versus filtering ones in terms of preventing the spread of Covid-19. Although surgical face masks, which have a maximum fixed price regulated by health administrations and are widely available, have poor peripheral sealing, systematic reviews and meta-analysis have failed to demonstrate that filtering face masks are better than surgical masks during influenza pandemics and SARS-CoV-2 [14, 15].

For the protection of healthcare professionals against the risks caused by biological agents, the European StandardisationOrganisations has defined requirements and tests methods for protective clothing against infective agents EN 14126:2004 [16]. Furthermore, the European Regulation (EU) 2016/425 [17] harmonizes the criteria for labeling and production of protective equipment, as well as analysis of products made in European countries or imported from third countries. In March 2020 the EU Commission made recommendations (EU) 2020/403 [18] that regulates market surveillance procedures for face masks used for the prevention and protection of harmful biological agents, within the context of the Covid-19 threat, in accordance with Council Directive 93/42 / EEC [19] and EU Regulation (EU) 2017/745 [20]. Regarding the Respiratory Protection Equipment (RPE) that health professionals must use, which includes both face masks and half masks based on European regulations, there are three types:

1. Surgical face masks: Respiratory masks considered a medical device (MD), which are classified into Type I, II and IIR, and are regulated by Standard EN 14683:2019+AC:2019 [21].
2. Filtering face masks: Respiratory masks which are considered PPE (not registered as a MD) and are classified into FFP1, FFP2 and FFP3, and regulated by the Standard EN 149:2001+A1:2009 [22].
3. Dual face masks: Respiratory masks that encompass the technical and functional properties of both surgical and filtering face masks.

It should be noted that in the USA, Occupational Safety and Health Administration (OSHA) obliges workers exposed to the inhalation of dangerous substances to pass an annual fit test on the safety in the use of filtering face masks as specified in OSHA 29 CFR 1910.134 [23], a test which is non-mandatory in the European Union.

The fabric used in surgical face masks is the only one that undergoes some form of quality control against biological hazards, contrary to PPE filtering face masks (FFP2 and FFP3), as stated in the directives that regulate them [21, 22]. Therefore, in order to be considered a dual face mask, filtering face masks should be used in conjunction with a surgical mask. There is reason to believe that this could compromise the user’s breathability, since the breathed air would have to pass through both fabrics. A previous study among healthcare providers wearing PPE masks during the 2003 severe acute respiratory distress syndrome (SARS) epidemic in Singapore reported new onset face mask‐associated headaches with a prevalence rate of 37.3% [24]. It can be speculated if these findings might be due to a slight hypoxemia.

**Fig 1:**
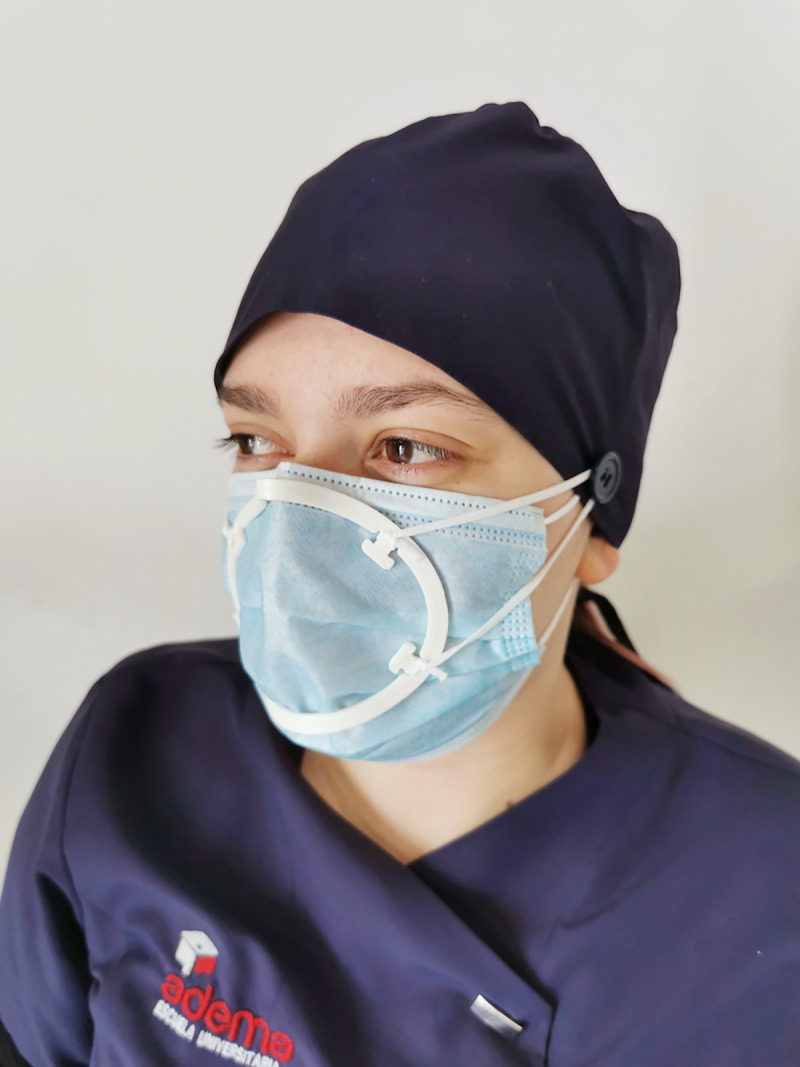
A health care professional using the personalized peripheral sealing device, designed at the ADEMA University School—UIB, with a surgical mask.

Considering this, the ADEMA University School–UIB has designed a personalized peripheral sealing device in the form of a ring based on thermoplastic polylactic acid (PLA), a resin obtained from corn starch, and which is suitable for healthcare use (Patent number P202030543). This device can be reused, it is biodegradable and can be disinfected with 0.1% sodium hypochlorite [25]. Although the device is produced following a standardized measurement, it can be adapted by digital pressure to the face of each individual by means of heat, thus becoming a personalized device (Fig 1). The use of such a custom-tailored sealing device would enable surgical face masks to become dual face masks, by reducing the risk of unfiltered breathed air leakage, conferring the characteristics of PPE and providing bidirectional patient-health professional protection, since it would be considered a medical device. This is of notable interest, especially when compared to the use of filtering face masks FFP2 and FFP3 used in industrial settings which come in standard sizes, and are designed using cephalometric patterns that in many cases differ from the morphogenetic typology of the professional or user.

In this sense, breathability would not be compromised with the use of custom-made surgical face masks with peripheral sealing, as these breathability tests are inherent and consistent with the characteristics of the standardized filtering fabric, which has also been tested against biological agents in a bidirectional manner.

The aim of the present study was to demonstrate that the use of the ADEMA- UIB personalized peripheral sealing device in surgical masks allows them to become dual face masks, by providing bidirectional protection to the professional or wearer, by means of the required tests for this verification. This includes carrying out studies in accordance with current European regulations on standardized materials, fit tests and terms of use. Since there are no regulatory standards in Europe that oblige workers or wearers of masks to perform fit tests, for this study we have applied OSHA Standard 29 CFR 1910.134 [23] with the purpose of following the protocol used in the USA to individually evaluate the respiratory protection equipment of workers. Furthermore, we have also considered that the device and face masks herein included must comply with the European Union standards EU 2020/403 [18], EN 14683:2019+AC: 2019 [21] and EN 149:2001+A1:2010 [22]. The null hypothesis tested here is that there are no differences in the distribution of the test result between the individuals that used the surgical mask without the peripheral sealing device (control group) and the individuals that used the surgical mask with the peripheral sealing device (intervention group).

## Materials and methods

The present study was approved by the Research Ethical Committee of the Balearic Islands (CEI-IB) (IB 4259—PI). The individual in this manuscript has given written informed consent (as outlined in PLOS consent form) to publish these case details.

### Manufacture of the thermoplastic device

**Fig 2:**
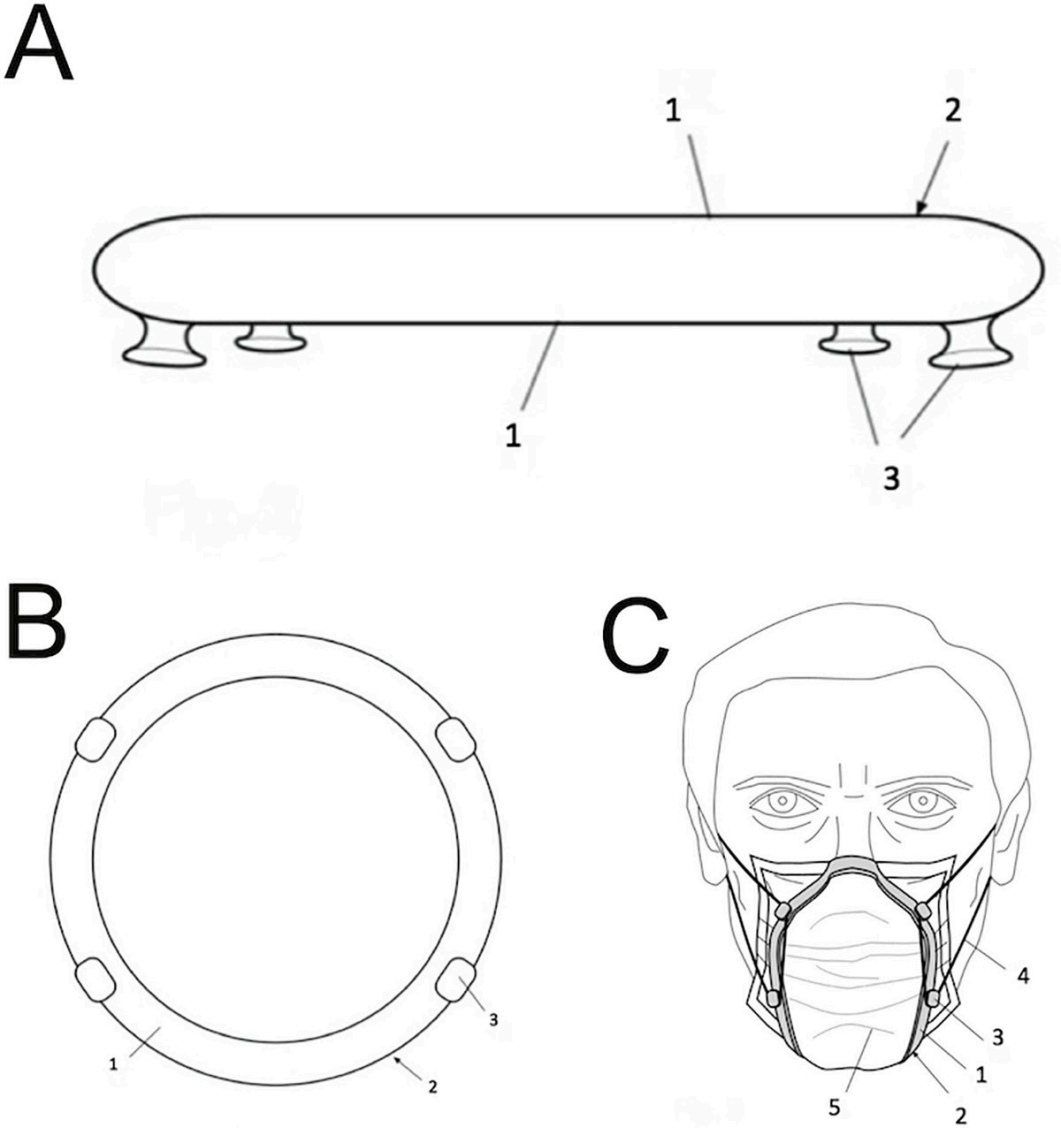
A. Representative image of the lateral view of the thermoplastic resin ring; B. Frontal view; and C. Representative image of the surgical mask sealed with the personalized peripheral sealing device, where 1: Thermoplastic resin ring; 2: Area of peripheral sealed; 3: Thermoplastic resin ring traction hook; 4: Elastic; 5: Surgical mask.

The ADEMA University School—UIB is an authorized institution for the manufacturing of customized sanitary and healthcare products, and carried out the design and manufacturing of the device in the form of a ring based on thermoplastic PLA resin. Initially, the device or ring has a standardized measurement of 13 cm (Fig 2). After its design with the Blender 3D software, and manufacturing by means of a 3D Print 4.0 printer (Kulzer Hanau, Germany), the thermoplastic ring is heated by immersing it in hot water at 60°C for 1–2 min. After, the device can be adapted by digital pressure to the face of each individual and thus adapts to their anatomical profile, and hardens by cooling at room temperature, which takes an estimated time of 15–20 seconds. After this procedure, the ring maintains the exact shape of the facial surface, resulting in a personalized device according to the anatomical characteristics of each individual. If the device is heated again, it loses its shape memory and can be readapted to the face of the same user or of any other, since it can be disinfected and sterilized if necessary. After molding the device, it can be adapted to the wearer using a surgical mask by attaching the mask elastic or an external elastic on the device’s hooks.

The design and validation of this device was carried out by means of a qualitative fit test and a quantitative fit test, as described below.

### Part 1: Qualitative fit test

To check the adjustment of the surgical face mask with the personalized peripheral sealing device, a sweet sensitivity test was performed with the 3M FT-10 Qualitative Fit-Test Kit (Saint Paul, Minnesota, USA), which complies with the current standards of OSHA [23]. The kit contains a test hood and collar, nebulizers and test solutions, which can be used to check the face-to-respirator seal on any particulate respirator or gas/vapor respirator with a particulate prefilter.

### Sample size calculation

A sample size calculation on the number of individuals per group was conducted a priori by estimating the expected effect size as 0.8from a previous pilot study. A 0.05 level of significance and a 85% contrast power value was used. Since the study involves two independent samples (control and intervention groups), the size of one group was fixed and the other group’s size was calculated. A large effect size was assumed (f = 0.8) and the size of one group was fixed as 30, resulting in a minimum of 29 individuals in each of the two groups.

#### Recruitment of participants

For this part of the study, a total of 117 individuals were selected. Inclusion criteria were as follows: people familiar with the use of face masks; absence of systemic diseases; aged between 18–65 years; absence of obvious facial hair; and signing the consent approved by the CEI-IB. People unfamiliar with the use of face masks, allergy or intolerance to sodium saccharin or to other materials used in the study, relevant systemic diseases, under 18 or over 65 years of age, presence of a beard, sideburns, a mustache or any obvious facial hair, pregnancy, and not signing the consent approved by the CEI-IB were excluded from the study.

Participants were recruited from different environments in order to attain diversity, while controlling for representativeness bias. They were selected from the ADEMA University School—UIB, nursing staff from the Infectious Diseases Service at Son Espases University Hospital, 112 Emergency Service of the Balearic Islands staff and teaching staff from the Miquel Porcel Educational Centre (Palma). Participants were assigned to one of the two groups (intervention or control group), after accepting their participation in the study voluntarily and freely by signing the consent form approved by the CEI-IB.

#### Sensitivity test

Before the fit test, a sensitivity test was performed to ensure that the individual was capable of detecting the sweet taste in minimal concentrations of sodium saccharin (the concentration of this solution is withheld as a trade secret by the producer company). For this, the test hood and collar were placed on the individual without a surgical mask, in such a manner that there was 15 cm between the individual’s face and the hood’s screen. Then, the individual was asked to breathe normally through the mouth with the tongue slightly extended and raise their hand when they noticed the sensitivity solution. The nebulizer was then held in a vertical position in front of the circular opening of the hood screen to dissipate the sweet mist inside, squeezing the nebulizer in sets of 10 (up to a maximum of 30 times), until the individual perceived the sweet solution in their oral cavity, following the manufacturer’s instructions of the3M FT-10 Qualitative Fit-Test Kit, which complies with OSHA current standards [23]. The number of nebulizer pulses needed was recorded for each individual, who then carried out water gargles and waited for 15 minutes before performing the fit test. Those who did not feel the sensitivity solution after 30 times were excluded from the study.

#### Fit test

For the fit test, the same technique as described in the sensitivity test was used, but using a concentrated sodium saccharin solution (83 g of USP sodium saccharin in 100 ml of water). The individuals were distributed as follows:

- Intervention group (IG): Surgical face mask with the PLA peripheral sealing device.
- Control group (CG): Surgical face mask without the peripheral sealing device.

**Fig 3:**
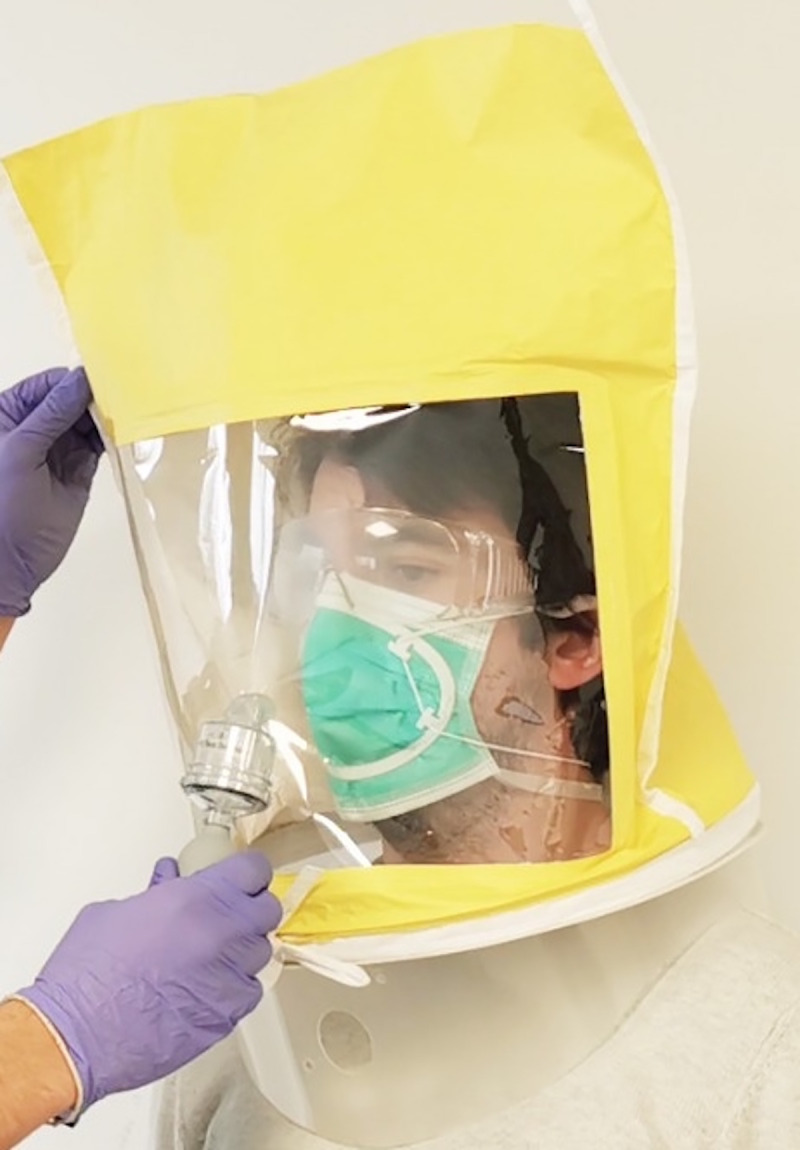
An individual of the intervention group being submitted to the qualitative fit test with the hood and nebulizer of the 3M FT-10 fit test.

All surgical face masks, with or without the device, were placed on the individual’s facial surface 5 minutes before the fit test to ensure its adjustment. The hood and the collar were placed on the individual as described in the sensitivity test and the nebulizer was pressed the same number of times as noted in the sensitivity test for that same individual. After 30 seconds, the nebulizer was squeezed half the amount of times and this procedure was repeated every 30 seconds until the end of the test, with a total duration of seven minutes (Fig 3).

Overall, during the qualitative fit test, a total of seven mobility exercises of 60 seconds each were performed, and the nebulizer was squeezed, every 30 seconds:

Phase 1 –Normal breathing
Phase 2 –Slow and deep breathing
Phase 3 –Breathing whilst moving the head to both sides
Phase 4 –Breathing whilst moving the head up and down
Phase 5 –Breathing spelling the alphabet
Phase 6 –Breathing whilst moving up and down from the waist
Phase 7 –Normal breathing

If the fit test was completed within 7 minutes without the individual having detected the sweet taste, the seal was considered optimal or adequate and the experiment was graded as such. If the subject detected the sweet test before seven minutes, the adjustment was considered inadequate and was scored as non-optimal, noting the time it took to detect the sweet taste as established in the protocol of the 3M FT-10 Fit test.

### Part 2: Quantitative fit test

This quantitative fit test quantified the leakage of ambient air into the facial mask used with the peripheral sealing device, with a specific atmosphere and expressed as a percentage of the total contaminated inhaled air. The measurement was carried out in an authorized laboratory (Textile Research Association (AITEX. Alcoi, Alicante, Spain)), an institution that validates PPE for professional use in Spain, in compliance with Standards EN 14683: 2019 + AC: 2019 [20] and EN 149: 2001 + A1: 2010 [21] of Regulation (EU) 2020/403 [17]. The percentage of total air leakage inhaled is the result of the specific particle filtration efficiency (PFE) of the tissue of the mask used (surgical mask Type II and IIR <2%, filtering mask FFP2 <8% and FFP3 <2%), to which the percentage of unfiltered inhaled air through the marginal mask mismatches in the test subject should be added.

#### Recruitment of participants

A total of 5 individuals were selected for this part of the study. AITEX establishes the inclusion and exclusion criteria for the subjects participating in the trial and the number of participants, who are qualified personnel from the institution itself.

#### Validation of the results

AITEX expedites an official report regarding the results of the tests in order to validate, where appropriate, whether the surgical face mask with the peripheral sealing device meets the requirements established in European regulations to be considered a PPE. For this, the results obtained with the combination of the surgical mask with peripheral sealing were compared with the amount of leakage obtained when FFP2 and FFP3 were used.

#### Fit test

**Fig 4:**
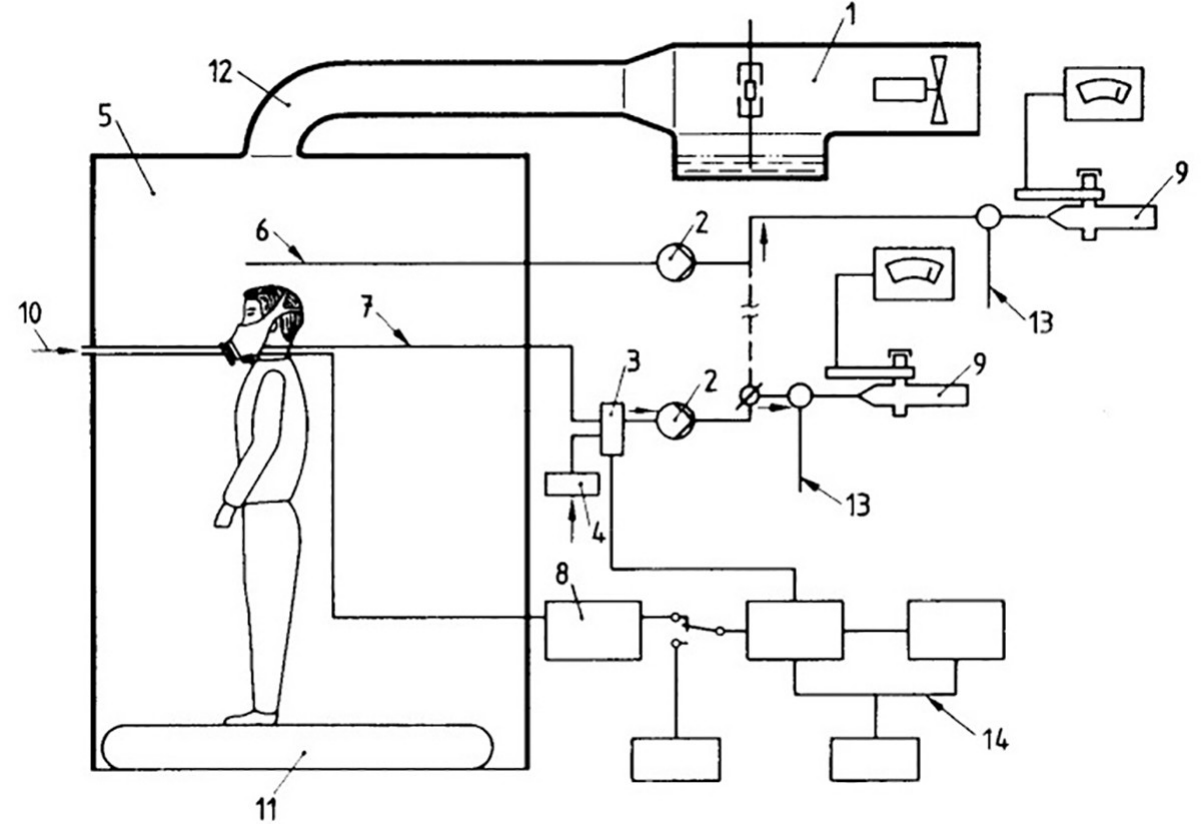
Representative image of the individual inside the cabin for the quantitative fit test obtained in the normativeEN 149:2001+A1:2009 [22], where 1. Atomizer; 2. Bomb; 3. Valve; 4. Filter; 5. Enclosure; 6. Sample extraction from enclosure; 7. Mask sample extraction; 8. Pressure gauge; 9. Photometer; 10. Surgical mask with the sealing device; 11. Continuous tape; 12. Duct and diffuser; 13. Additional air; 14. Intermittent interface for sample extraction; 15. Exhalation.

The quantitative inward leakage test is performed using an aerosol of 2% sodium chloride (NaCl) in distilled water, in a watertight cabinet with probes sensitive to this substance designed for this purpose. This saline atmosphere enters in the cabin through a flow distributor at a minimum velocity of 0.12 m/s. The air inside the face mask used is sampled and analyzed during the inhalation phase of the respiratory cycle to determine the NaCl content. The sample is drawn by making a hole in the face mask and inserting a probe through which the sample is drawn. Variation of pressure within the surgical face mask is used to operate an exchange valve so that only inhaled air is sampled (Fig 4).

The test sequence is as follows:

- The surgical face mask with the peripheral sealing device is placed on the subject 5 minutes before the fit test to ensure its correct position.
- The subject walks at a speed of 6 km/h for 2 minutes and the concentration of the test solution inside the surgical mask is measured to establish the background level and obtain a stable reference reading.
- The test atmosphere is activated with a 2% NaCl solution and while walking, the test subject performs five preset exercises and the concentration of the solution in the cabin and the leakage into the surgical face mask is recorded in each exercise.
- The percentage of total air inhaled will determine whether the test has been satisfactory or not.

### Statistical analysis

Two independent samples were analyzed after assigning the subjects to one of the study groups:

- Individuals that carried out the test with a surgical face mask without the peripheral sealing device (Control).
- Individuals that carried out the test with a surgical face mask with the peripheral sealing device (Intervention).

A Fisher’s exact test was used for proportion comparisons. Continuous variables were examined for normality by Kolmogorov-Smirnov-Lilliefors test, and comparisons between two groups were conducted by using either a Student’s *t*-test or the Mann-Whitney U test when appropriate. All calculations were performed using the free software R. Statistical significance was considered when *p*≤0.05.

## Results

### Part 1: Qualitative fit test

**Fig 5:**
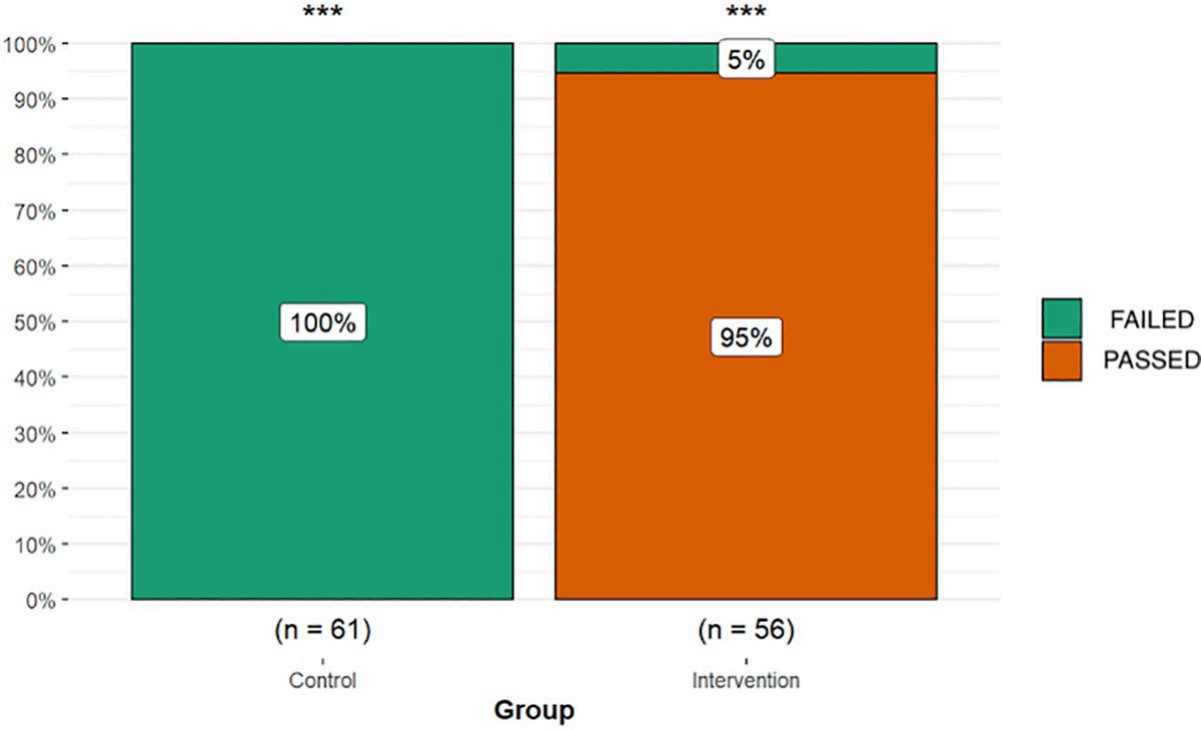
Distribution of the test result in the control and intervention groups. The PASSED percentage refers to the individuals that optimally passed the test (7 minutes without feeling the sweet taste). The FAILED percentage refers to the individuals that felt the sweet taste at some point of the experiment.

Once the comparability of the two groups was proved, the distribution of PASSED (those who optimally passed the test, by not sensing the sweet test after 7 minutes) with that of FAILED (those who felt the sweet taste before 7 minutes) was compared (Fig 5).

It is important to consider that out of the n_i_ = 56 volunteers who did the test with the device, 53 did not feel the taste (95%), whereas all of the n_c_ = 61 volunteers who performed the test without the device felt it. In this case, the Fisher’s exact test of proportions comparison resulted in a *p<*2.2·10^−16^, *CI*_95%_ = (203 *∞*).

An χ^2^ test was performed to analyze the homogeneity in the distribution of the response between the two groups, resulting in a Cramer coefficient = 0.95 [*CI*_95%_ = (0.85,1.02)], thus rejecting the null hypothesis that there are no differences in the distribution of the test result between the Control group and the Intervention group (*p* = 9.3·10^−25^). The power of the contrast, for a significance level of 0.05, is 100%.

#### Characteristics and results obtained in the intervention group

A total of 56 individuals were initially part of the Intervention group, with the age ranging between 18 and 53 years old, with a mean age of 26,2 years. Of these, three individuals were not able to feel the sweet taste in the sensitivity test and thus were excluded from the study. Out of the 28 male participants, 27 passed the qualitative test optimally (7 minutes without feeling the sweet taste) according to the OSHA guidelines [22] and only one individual felt the sweet taste at 60s. Out of the 31 women, 29 passed the test optimally (7 minutes without feeling the sweet taste) and only two felt the sweet taste at 1min 50s and 3min and 9s. Overall, 53 subjects (95%) passed the test optimally (7 minutes without feeling the sweet taste) and three (5%) felt the sweet taste in the time interval between 60s and 3min 9s, with a mean of 120s (Table 1).

**Table 1:** Number and percentage of individuals (intervention and control groups) that optimally passed the qualitative fit test.

Group	Intervention		Control	
Gender	Optimally passed the fit test	Failed the Fit Test	Optimally passed the fit test	Failed the Fit Test
**Male**	24	1	0	28
**Female**	29	2	0	33
**Total**	53	3	0	61
**Optimally passed (%)**	95%		0.00%	

#### Characteristics and results obtained in the control group

A total of 61 individuals were initially assigned to the Control group, with the age ranging between 18 and 61 years old, with a mean age of 33,7 years. Of these, four individuals were not able to feel the sweet taste in the sensitivity test and were excluded from the study. None of them passed the qualitative test (0.00%). The sweet taste was detected, for all participants, between 8s and 1min 55s, with a mean time of 18s (Table 1).

#### Variables comparison

*a) Comparison according to gender*. There were 28 individuals of the male gender and 33 of the female gender in the control group, while in the Intervention group there were 25 individuals of the male gender and 31 of the female gender. A Fisher’s exact test was used for the comparisons of proportions. There were no significant differences between the proportion of either men or women among groups (Table 2).

**Fig 6:**
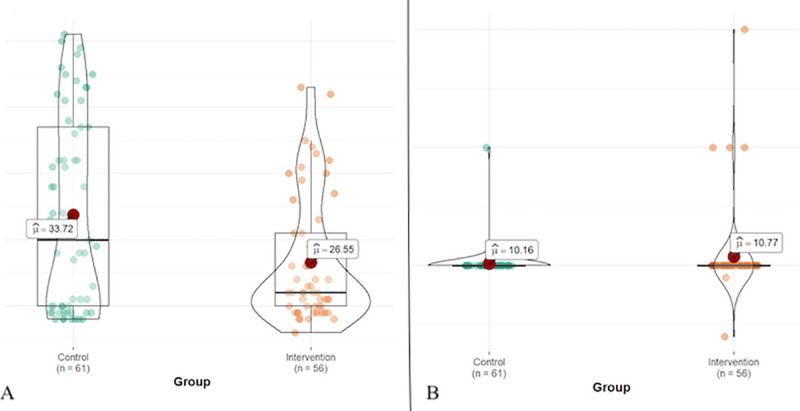
A. Distribution of the individuals of the study by age in the Control and Intervention groups; B. Distribution of the number of activations in the sensitivity test in the Control and Intervention groups.

*b) Comparison according to age*. Age was not normally distributed throughout the groups, so a comparison of medians was made using the Mann-Whitney test. From the point estimate of the test statistic (0.18) and the confidence interval, no significant differences were observed in the medians of the ages of both groups (*p* = 0.051) (Fig 6).

*c) Comparison of the number of activations of the nebulizer per unit of time in the sensitivity test*. The number of activations in the initial sensitivity test was not normally distributed in the groups, so a comparison of medians was made using the Mann-Whitney test. As was the case for age, no significant differences were observed in the medians of the number of activations of both groups [r = -0.04, CI95% = (-0.24,0.14) p-valor = 0.652] (Fig 6).

**Table 2:** Comparison of proportions by gender.

Gender	Intervention Group (n -%)	Control group (n -%)	*P* value CI (95%)	Odds ratio
**Male**	25–45%	28–46%	1	(0.4 2.3)
**Female**	31–55%	33–54%	1	(0.43 2.1)

### Part 2: Quantitative fit test

The mean percentage of air leakage into the surgical face mask with the peripheral sealing device, used by the 5 subjects who underwent the fit test, was 7.02% (F). Thus, as it exceeds the requirements established in European regulations, the surgical face mask with the peripheral sealing device was certified by AITEX as a PPE (Report number 2021EC0016).

**Fig 7:**
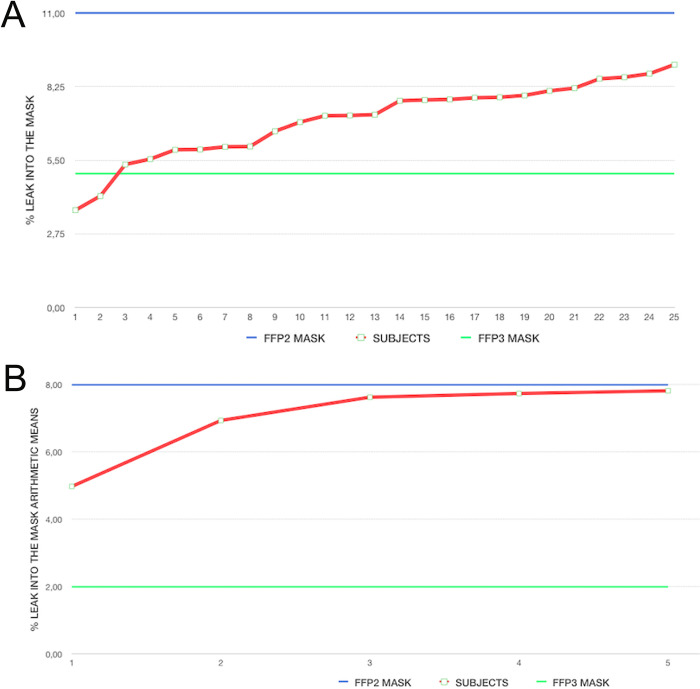
A. Arithmetic means of individual wearers for total inward leakage, between 4.98% and 7.82%, values lower than those established by the FFP2 filter masks (<8.00%) and higher than those of the FFP3 (<2%); B. Individual exercise results for total inward leakage (i.e. 10 subjects x 5exercises), expressed as a percentage of total air inhaled, between 3.63% and 9.07%, values between those established for filter masks FFP2 (<11%) and higher than those for FFP3 (<5%).

Table 3 describes the individual exercises for total inward leakage (10 subjects x 5 exercises) expressed as a percentage of total air inhaled. The results range between 3.63% and 9.07%, values which are lower than those established for filtering masks FFP2 (<11%) and higher than those of the FFP3 (<5%) which have been anthropometrically fitted to the subjects (Fig 7).

**Table 3:** Percentage of the total inward leakage in each one of the 5 individuals in the 5 different exercises.

Percentage of leakage into the masks			
Subjects and exercise	FFP2 masks	SM+PSD	FFP3 masks
**1**	11.00	3.63	5.0
**2**	11.00	4.16	5.0
**3**	11.00	5.34	5.0
**4**	11.00	5.54	5.0
**5**	11.00	5.89	5.0
**6**	11.00	5.9	5.0
**7**	11.00	6.00	5.0
**8**	11.00	6.01	5.0
**9**	11.00	6.58	5.0
**10**	11.00	6.92	5.0
**11**	11.00	7.16	5.0
**12**	11.00	7.17	5.0
**13**	11.00	7.20	5.0
**14**	11.00	7.72	5.0
**15**	11.00	7.75	5.0
**16**	11.00	7.77	5.0
**17**	11.00	7.83	5.0
**18**	11.00	7.85	5.0
**19**	11.00	7.92	5.0
**20**	11.00	8.09	5.0
**21**	11.00	8.19	5.0
**22**	11.00	8.54	5.0
**23**	11.00	8.60	5.0
**24**	11.00	8.73	5.0
**25**	11.00	9.07	5.0

SM+PSD: surgical mask with the peripheral sealing device.

The arithmetic mean of the individual carriers for the total inward leakage can be seen in Table 4, whereby it ranges between 4.98% and 7.82%, values which are lower than those established by the FFP2 filter masks (<8.00%) and higher than those of the FFP3 (<2%) which have been anthropometrically fitted to the subjects (Fig 7).

**Table 4:** Mean percentage of ambient air leakage into the masks in the 5 subjects that participated in the quantitative test.

Percentage of Leakage into the face mask—Arithmetic Means			
Subjects	FFP2 mask	SM+PSD	FFP3 mask
**1**	8.00	4.98	2.0
**2**	8.00	6.94	2.0
**3**	8.00	7.63	2.0
**4**	8.00	7.74	2.0
**5**	8.00	7.82	2.0
**Total Mean**	8.00	7.02	2.0

SM+PSD: surgical mask with the peripheral sealing device.

This quantitative inward leakage test only validates the use of surgical masks with the peripheral sealing device as a PPE in ambient temperature situations. The device did not pass the test at 70°C, due to the thermoplastic characteristics of the PLA. The peripheral sealing device did not exceed the IL_46/50_ (Inward Leakage exceeded in 46 out of 50 individual exercises) and the TIL_8/10_ (Total Inward Leakage exceeded in 8 out of 10 wearers) in arithmetic means at 70°, which are extreme conditions only necessary for industrial use.

## Discussion

Recent studies on the efficacy of face masks in the prevention of airborne transmission of SARS-CoV-2 [26] demonstrate the importance of their peripheral sealing to favor that the air we breathe is filtered in its entirety and efficiently by the tissues that compose it. Therefore, they require optimal anatomical adaptation, which is difficult to achieve with standard filtering masks that have been designed with predetermined facial patterns, or with surgical masks that, despite being medical devices that offer biological filtration efficiency (BFE), lack peripheral sealing. In this sense, here we propose the use of a peripheral sealing device, devised by the ADEMA University School–UIB, which achieves a seal with a marginal mismatch in only 5% of users when used with type II and IIR surgical masks. This mismatch is much lower than that observed in filtering masks when subjected to qualitative tests, ranging between 86.4% and 80.5% [13, 27], thus conferring certain dual properties to surgical masks, by providing bidirectional protection to the user thanks to an efficient peripheral adjustment.

A comprehensive review of mask filtration measures has recently been published [28], as well as a meta-analysis showing the utility of their use to prevent Covid-19 transmission [29]. The results of these studies showed that fit tests carried out in face masks determine not only the protection of the professionals or users who use them, but also highlight the routes of infection of diseases such as Covid-19, such as the transmission by aerosols in closed places where the air is not properly renewed [30]. The breathability index, together with the fit test results of the face masks, can be of great relevance, since they can both condition the infection routes of aerosols.

Currently, the lack of peripheral adjustment of surgical face masks has led to the massive use of filtering masks FFP2 or FFP3, especially in health facilities, due to their better marginal adjustment. However, it is important to emphasize that besides the adjustment of the mask to the wearer’s face, the protection a mask can offer will also significantly vary based on the particle filtration efficiency and the BFE [31–33]. FFP masks have a high level of breathability, which means that, during inhalation and exhalation, the dense tissue of these masks will hind the free passage of air, favoring the passage of this air through marginal mismatches where the differential pressure is lower. This is the reason why the main use of FFP filtering masks, up until the pandemic, has been for the protection of workers in situations where industrial amounts of dust are generated. These special circumstances of exposure are those that condition a respiratory protection fabric with a high breathability index of these FFP masks, which are not suitable in other situations, such as health clinics, education centers or other closed places. In such conditions, the unfiltered air during expiration is what causes contaminated aerosols in closed spaces. This high breathability index explains why FFP masks are not the appropriate ones for health clinics or other closed and crowded environments [34–36].

Thus, surgical masks can be considered suitable because of their lower breathability index compared to filter masks, and are thus considered a respiratory protection equipment with BFE.

Furthermore, the health administrations of some countries recommend the use of filtering face masks FFP2 or FFP3 simultaneously with type II or IIR surgical face masks, to protect professionals in high-risk situations against infectious agents [37, 38], especially in the current Covid-19 pandemic. However, this simultaneous use could compromise the breathability parameters, established in the standards that regulate FFP and surgical masks, EN 14683:2019+AC:2019 [20] and EN 149:2001+A1:2009 [22] respectively, by affecting the flow of breathed air through the fabric of both masks, which could leak through the peripheral spaces surrounding the mask, since the pressure gradient between the inside and outside of the masks is much higher than that established when face masks are used individually [34–36].

Thus, the use of type II or IIR surgical masks together with the personalized peripheral sealing device would accomplish the specifications established in the European standards EU 2020/403 [18], EN 14683: 2019 + AC: 2019 [21] and EN 149: 2001 + A1: 2010 [22], providing an adequate peripheral sealing, as shown in the present study, and an optimal bacterial and particle filtration efficiency. These last mentioned characteristics were also proved in the present work, endorsed by the quantitative fit test performed by AITEX, an authorized center to approve masks as PPE through quantitative testing of inhaled air leakage into the interior. Since the combination of a surgical mask and the peripheral sealing device achieved a leak of only 6.5%, it was considered optimal. Importantly, it improved the efficiency observed in filtering face masks with prior anthropometric adjustment, including FFP1 (<25%) and FFP2 (<11%), and was very close to those achieved with FFP3 (<5%).

Making face masks with bi-directional dual protection properties available to professionals who work in high-risk situations of Covid-19 infection allows them to safely carry out their work with infected patients and in closed environments, where there is little air renewal and a large influx of people, such as in health and educational centers or public transport, among others. These are situations in which it has recently been shown that maintaining a safe distance is not enough [39], with a higher probability of infection by aerosols present in the environment. This demonstrates the aerial transmission of Covid-19 and why two meters of interpersonal distance is not enough in these situations [40]. Thus, an optimal peripheral sealing of surgical masks should also be required in compromised clinical situations where the equipment used generates aerosols from patients, such as dental clinics, which is why it is necessary to apply effective measures to control drops and aerosols, especially during this current pandemic caused by Covid-19 [41].

This study does not analyze or question the technical characteristics of masks. Its sole objective was to find an optimal seal for this type of surgical mask using a custom peripheral adjustment device.

The use of face masks to avoid contagion against Covid-19 is necessary, although the fit and characteristics of the materials they are made of must be adequate. There is scientific evidence that confirms that these masks are not being used correctly by health professionals, who continue to be infected by Covid-19. We believe, therefore, that it is necessary to carry out new studies to achieve new designs and more suitable materials for face masks to achieve respiratory protection for healthcare professionals in risk situations against Covid-19 and other infectious processes.

## Conclusions

The use of a personal PLA thermoplastic peripheral sealing device in conjunction with surgical masks provides a seal with a marginal discrepancy in 5% of users in the qualitative fit test and an internal leakage of only 7% in the quantitative fit test. The improved seal achieved by using this PSD together with a surgical mask can be attributed to the personal character of the device and that it is individually customized. The findings of the study thus confirm that it is possible to achieve an improved peripheral sealing of surgical masks without compromising the users breathability. The present study may contribute to a better protection against infection by COVID-19 and other pathogens and should suit the category of Personal Protective Equipment.

## Supporting information

S1 File Qualitative analysis results. Table with the official results of the qualitative fit test of the Intervention Group (Mask with the Peripheral Sealing Device) and the Control Group (Mask without the Peripheral Sealing Device).
(XLSX)

S2 File Qualitative test: Statistical analysis. Complete statistical analysis description of the Qualitative Fit test.
(PDF)

S3 File Results analysis—quantitative test. Tables and Graphs of the Quantitative Fit test.
(XLSX)

## References

[1] World Health Organization Novel Coronavirus (2019-nCoV) situation reports. who.int [Internet]. Geneva: World Health Organization; 2020 [updated 2020; cited 23 Mar 2020]. Available from: https://www.who.int/emergencies/diseases/novel-coronavirus-2019/situation-reports

[2] Friese CR, Veenema TG, Johnson JS, Jayaraman S, Chang JC, Clever LH. Respiratory Protection Considerations for Healthcare Workers During the COVID-19 Pandemic. Health Secur. 2020;18:237–240. doi: 10.1089/hs.2020.0036 32320327 PMC7360110

[3] Garcia Godoy LR, Jones AE, Anderson TN, Fisher CL, Seeley KML, Beeson EA, et al. Facial protection for healthcare workers during pandemics: a scoping review. BMJ Glob Health. 2020; 5:e002553. doi: 10.1136/bmjgh-2020-002553 32371574 PMC7228486

[4] WHO Director General’s opening remarks at the media briefing on 2019 novel coronavirus, 2020. who.int [Internet]. Geneva: World Health Organization; 2020 [updated 2020; cited 23 Mar 2020]. Available from: https://www.who.int/dg/speeches/detail/who-director-general-s-opening-remarks-at-the-media-briefing-on-2019-novel-coronavirus-7-february-2020.

[5] Shah AS, Tande AJ, Challener DW, O’Horo JC, Binnicker MJ, Berbari EF. Diagnostic Stewardship: An Essential Element in a Rapidly Evolving COVID-19 Pandemic. Mayo Clin Proc 2020; 95:S17–S19. doi: 10.1016/j.mayocp.2020.05.039 32807516 PMC7309714

[6] Nguyen LH, Drew DA, Graham MS, Joshi AD, Guo CG, Ma W, et al. Risk of COVID-19 among front-line health-care workers and the general community: a prospective cohort study. Lancet Public Health. 2020; 5:e475–e483. doi: 10.1016/S2468-2667(20)30164-X 32745512 PMC7491202

[7] OSHA. Guidance on Preparing Workplaces for COVID-19. osha.gov [Internet]. Washington DC: Occupational Safety and Health Act; 2020 [updated 01 March 2020, cited 20 Dec 2020]. Available from: https://www.osha.gov/Publications/OSHA3990.pdf

[8] Meng L, Hua F, Bian Z. Coronavirus Disease 2019 (COVID-19): Emerging and Future Challenges for Dental and Oral Medicine. J Dent Res. 2020; 99:481–7. doi: 10.1177/0022034520914246 32162995 PMC7140973

[9] National Center for Immunization and Respiratory Diseases, Division of Viral Diseases. Interim Infection Prevention and Control Recommendations for Patients with Suspected or Confirmed Coronavirus Disease 2019 (COVID-19) in Healthcare Settings. cdc.gov [Internet]. Washington: Centers for Disease control and Prevention [updated 12 Dec 2019; cited 14 Dec 2020]. Available from: https://www.cdc.gov/coronavirus/2019-ncov/infection-control/control-recommendations.html

[10] Peng X, Xu X, Li Y, Cheng L, Zhou X, Ren B. Transmission routes of 2019-nCoV and controls in dental practice. Int J Oral Sci. 2020;12:9. doi: 10.1038/s41368-020-0075-9 32127517 PMC7054527

[11] Prati C, Pelliccioni GA, Sambri V, Chersoni S, Gandolfi MG. COVID-19: its impact on dental schools in Italy, clinical problems in endodontic therapy and general considerations. Int Endod J. 2020; 53:723–725. doi: 10.1111/iej.13291 32277770 PMC7262194

[12] Wen Z, Yu L, Yang W, Hu L, Li N, Wang J, et al. Assessment the protection performance of different level personal respiratory protection masks against viral aerosol. Aerobiologia 2013; 29:365–372. doi: 10.1007/s10453-012-9286-7 32214627 PMC7087618

[13] Eficacia de la utilización de los equipos de protección respiratoria. Evaluación cuantitativa del ajuste facial en mascarillas autofiltrantes. 2011. [Efficacy of the use of respiratory protection equipment. Quantitative evaluation of facial adjustment in self-filtering masks. 2011] Spanish.fremap.es [Internet]. Madrid: FREMAP; 2008 [updated 11 Dec 2008; cited 28 Dec 2020]. Available from:https://prevencion.fremap.es/Documentos%20observatorio%20siniestralidad/Estudio%20eficacia%20equipos%20proteccion%20respiratoria.pdf

[14] Long Y, Hu T, Liu L, Chen R, Guo Q, Yang L, et al. Effectiveness of N95 respirators versus surgical masks against influenza: A systematic review and meta-analysis. J Evid Based Med 2020; 13:93–101. doi: 10.1111/jebm.12381 32167245 PMC7228345

[15] Barycka K, Szarpak L, Filipiak KJ, Jaguszewski M, Smereka J, Ladny JR, et al. Comparative effectiveness of N95 respirators and surgical/face masks in preventing airborne infections in the era of SARS-CoV2 pandemic: A meta-analysis of randomized trials. PLoS One. 2020;15:e0242901. doi: 10.1371/journal.pone.0242901 33320847 PMC7737973

[16] EN 14126:2003. Protective clothing including hand and arm protection and lifejackets. standards.cen.eu [Internet]. Brussels: European Committee for Standardization; 2014 [updated 20 Feb 2014; cited 28 Dec 2020]. Available from: https://standards.cen.eu/dyn/www/f?p=204:110:0::::FSP_PROJECT,FSP_ORG_ID:6634,6143&cs=1762F21421CCAB5CDFA53171933C67440

[17] Regulation (EU) 2016/425 of the European Parliament and of the Council of 9 March 2016 on personal protective equipment and repealing Council Directive 89/686/EEC.eur-lex-europa.eu [Internet]. Brussels: Parlamento Europeo. Consejo Europeo; 2013 [updated 01 Jul 2013; cited 28 Dec 2020]. Available from: https://eur-lex.europa.eu/legal-content/ES/TXT/?uri=CELEX%3A32016R0425

[18] Commission Recommendation (EU) 2020/403 of 13 March 2020 on conformity assessment and market surveillance procedures within the context of the COVID-19 threat.eur-lex-europa.eu [Internet]. Brussels: Comisión Europea; 2013 [updated 01 Jul 2013; cited 28 Dec 2020]. Available from: https://eur-lex.europa.eu/legal-content/ES/TXT/?uri=CELEX:32020H0403

[19] Council Directive 93/42/EEC of 14 June 1993 concerning medical devices. eur-lex-europa.eu [Internet]. Brussels: Comisión Europea; 2007 [updated 11 Oct 2007; cited 28 Dec 2020]. Available from: https://eur-lex.europa.eu/eli/dir/1993/42/oj/spa

[20] Regulation (EU) 2017/745 of the European Parliament and of the Council of 5 April 2017 on medical devices, amending Directive 2001/83/EC, Regulation (EC) No 178/2002 and Regulation (EC) No 1223/2009 and repealing Council Directives 90/385/EEC and 93/42/EEC.eur-lex-europa.eu [Internet]. Brussels: Parlamento Europeo. Consejo Europeo; 2013 [updated 01 Jul 2013; cited 28 Dec 2020]. Available from: https://eur-lex.europa.eu/legal-content/ES/TXT/?uri=CELEX%3A32017R0745

[21] EN 14683:2019+AC:2019. Medical face masks—Requirements and test methods. standards.cen.eu [Internet]. Brussels: European Committee for Standardization; 2019 [updated 07 Aug 2019; cited 28 Dec 2020]. Available from: https://standards.cen.eu/dyn/www/f?p=204:110:0::::FSP_PROJECT:69675&cs=1956C06A1BAF887FF462DD56057D34F29

[22] EN 149:2001+A1:2009. Respiratory protective devices—Filtering half masks to protect against particles—Requirements, testing, marking. standards.cen.eu [Internet]. Brussels: European Committee for Standardization; 2009 [updated 06 May 2009; cited 28 Dec 2020]. Available from: https://standards.cen.eu/dyn/www/f?p=204:110:0::::FSP_PROJECT,FSP_ORG_ID:32928,6062&cs=1FC98AD34A5EE26A0CB5A6155ED4D6E5E

[23] Occupational Safety and Health Administration. OSHA 29 CFR 1910.134. osha.gov [Internet]. Washington DC: Occupational Safety and Health Act; 2006 [updated 08 Jun 2011, cited 20 Dec 2020]. Available from: https://www.osha.gov/laws-regs/regulations/standardnumber/1910/1910.134

[24] Lim EC, Seet RC, Lee KH, Wilder-Smith EP, Chuah BY, Ong BK. Headaches and the N95 face-mask amongst healthcare providers. Acta Neurol Scand. 2006; 113:199–202. doi: 10.1111/j.1600-0404.2005.00560.x 16441251 PMC7159726

[25] Kampf G, Todt D, Pfaender S, Steinmann E. Persistence of coronaviruses on inanimate surfaces and their inactivation with biocidal agents. J Hosp Infect. 2020;104:246–251. doi: 10.1016/j.jhin.2020.01.022 32563551 PMC7834866

[26] Ueki H, Furusawa Y, Iwatsuki-Horimoto K, Imai M, Kabata H, Nishimura H, et al. Effectiveness of Face Masks in Preventing Airborne Transmission of SARS-CoV-2. mSphere. 2020; 5:e00637–20. doi: 10.1128/mSphere.00637-20 33087517 PMC7580955

[27] Lee SA, Hwang DC, Li HY, Tsai CF, Chen CW, Chen JK. Particle Size-Selective Assessment of Protection of European Standard FFP Respirators and Surgical Masks against Particles-Tested with Human Subjects. J Healthc Eng. 2016; 2016:8572493. doi: 10.1155/2016/8572493 27195721 PMC5058571

[28] Clase CM, Fu EL, Ashur A, Beale RCL, Clase IA, Dolovich MB, et al. Forgotten Technology in the COVID-19 Pandemic: Filtration Properties of Cloth and Cloth Masks-A Narrative Review. Mayo Clin Proc. 2020 Oct;95(10):2204–2224. doi: 10.1016/j.mayocp.2020.07.020 33012350 PMC7834536

[29] Li Y, Liang M, Gao L, Ayaz Ahmed M, Uy JP, et al. Face masks to prevent transmission of COVID-19: A systematic review and meta-analysis. Am J Infect Control. 2020: S0196-6553(20)31043-9. doi: 10.1016/j.ajic.2020.12.007 33347937 PMC7748970

[30] Setti L, Passarini F, De Gennaro G, Barbieri P, Perrone MG, Borelli M, et al. Airborne Transmission Route of COVID-19: Why 2 Meters/6 Feet of Inter-Personal Distance Could Not Be Enough. Int J Environ Res Public Health. 2020;17:2932. doi: 10.3390/ijerph17082932 32340347 PMC7215485

[31] van der Sande M, Teunis P, Sabel R. Professional and home-made face masks reduce exposure to respiratory infections among the general population. PLOS ONE, 2008; 3:e2618, 2008. doi: 10.1371/journal.pone.0002618 18612429 PMC2440799

[32] Hill WC, Hull MS, MacCuspie RI. Testing of Commercial Masks and Respirators and Cotton Mask Insert Materials using SARS-CoV-2 Virion-Sized Particulates: Comparison of Ideal Aerosol Filtration Efficiency versus Fitted Filtration Efficiency. Nano Lett. 2020; 20:7642–7647. doi: 10.1021/acs.nanolett.0c03182 32986441 PMC7534799

[33] Mueller AV, Eden MJ, Oakes JM, Bellini C, Fernandez LA. Quantitative Method for Comparative Assessment of Particle Removal Efficiency of Fabric Masks as Alternatives to Standard Surgical Masks for PPE. Matter. 2020; 3:950–962. doi: 10.1016/j.matt.2020.07.006 32838296 PMC7346791

[34] Rosner E. Adverse effects of prolonged mask use among healthcare professionals during COVID‐19. J Infect Dis Epidemiol 2020; 6:130. doi: 10.23937/2474-3658/1510130

[35] Ong JJY, Bharatendu C, Goh Y, et al. Headaches associated with personal protective equipment—a cross‐sectional study among frontline healthcare workers during COVID‐19. Headache 2020; 60:864–877. doi: 10.1111/head.13811 32232837

[36] Rothamer DA, Sanders S, Reindl D, Bertram TH. Strategies to minimize SARS-COV-2 transmission in classroom settings: Combined impacts of ventilation and mask effective filtration efficiency. University of Winconsin-Madison. Published December 31, 2020. Posted January 4, 2021. Preprint 10.1101/2020.12.31.20249101.

[37] Roberge RJ. Effect of surgical masks worn concurrently over N95 filtering facepiece respirators: extended service life versus increased user burden. J Public Health Manag Pract. 2008;14:E19–26. doi: 10.1097/01.PHH.0000311904.41691.fd 18287908

[38] Ahmad F, Hasan SM, Rai S. Face Masks—Rationale in Prevention of Respiratory Viral Epidemic (COVID-19) International Journal of Experimental Dental Science 2020; doi: 10.5005/jp-journals-10029-1208

[39] Sommerstein R, Fux CA, Vuichard-Gysin D, Abbas M, Marschall J, Balmelli C, et al. Risk of SARS-CoV-2 transmission by aerosols, the rational use of masks, and protection of healthcare workers from COVID-19. Antimicrob Resist Infect Control. 2020; 9:100. doi: 10.1186/s13756-020-00763-0 32631450 PMC7336106

[40] Setti L, Passarini F, De Gennaro G, Barbieri P, Perrone MG, Borelli M, et al. Airborne Transmission Route of COVID-19: Why 2 Meters/6 Feet of Inter-Personal Distance Could Not Be Enough. Int J Environ Res Public Health. 2020;17:2932. doi: 10.3390/ijerph17082932 32340347 PMC7215485

[41] Yue L. Ventilation in the Dental Clinic: An Effective Measure to Control Droplets and Aerosols during the Coronavirus Pandemic and Beyond. Chin J Dent Res. 2020;23:105–107. doi: 10.3290/j.cjdr.a44746 32548601

