# Supplementary material for: Study of the use of a personalized peripheral sealing device on surgical face masks in high-risk situations against COVID-19
Source: PLoS One. 2021 Aug 6;16(8):e0253382. doi: 10.1371/journal.pone.0253382 (PMC8345852; doi:10.1371/journal.pone.0253382)
Supplement: S2 File — Complete statistical analysis description of the Qualitative Fit test. (PDF) [file pone.0253382.s002.pdf]

Augmented data

Juan Jiménez Recaredo

10/4/2021

Resumen:

Se trata de dos muestras independientes: una con voluntarios que utilizaron la mascarilla **sin** el dispositivo (Controles) y otra con voluntarios que realizaron la prueba **con** el dispositivo diseñado en ADEMA (Intervención).

Los grupos son homogéneos en la distribución de hombres y mujeres, sin que haya podido encontrar diferencias estadísticamente significativas en las medianas de las edades ni en las medianas de la sensibilidad al olor, medida según el número de activaciones en el test inicial.

La proporción de voluntarios del grupo intervención que no detecta ningún olor es significativamente mayor que la del grupo control ($p\text{-valor}=9.3\cdot 10^{-25}$), para una potencia del contraste = 100%

Comparación de los grupos:

Como paso inicial hay que asegurar que los grupos (después de eliminar del análisis los voluntarios que no fueron evaluados en la prueba) son comparables en cuanto a las variables características de cada uno: 1) las distribuciones de hombres y mujeres, 2) las de las edades y 3) la sensibilidad inicial medida por el número de activaciones.

Comparación por Género

La comparación de las distribuciones del Género según el grupo se

puede hacer, o bien comparando las proporciones apropiadas (que fue lo que presenté en el análisis anterior), ó bien se pueden hacer los test de homogeneidad \(\chi^2\).

En el primer caso, tomando en cuenta que luego de las modificaciones hechas el grupo Control está compuesto por 28 hombres y 33 mujeres, mientras que en el grupo Intervención hay 25 hombres y 31 mujeres, los resultados de las comparaciones de proporciones usando el test exacto de Fisher (este es el test que debe ser usado porque las muestras son pequeñas) son:

**Variable**	**Control**	**Intervention**	**p-valor**	**CI(95%) (for the Odds ratio)**
Males (totals and %)	28, 46%	25, 45%	1	(0.48 2.3)
Females (totals and %)	33, 54%	31, 55%	1	(0.43 2.1)

De manera que no hay diferencias significativas entre la proporción de hombres en el grupo control y la proporción en el grupo intervención. Lo mismo vale para la comparación de proporciones de mujeres entre ambos grupos.

Los detalles son:

```
Tabla.h=rbind(c(28,33),c(25,31))
fisher.test(Tabla.h, alternative="two.sided")
```

```
##
##  Fisher's Exact Test for Count Data
##
## data:  Tabla.h
## p-value = 1
## alternative hypothesis: true odds ratio is not equal
to 1
## 95 percent confidence interval:
##  0.4767101 2.3246637
```

```
## sample estimates:
## odds ratio
##   1.051632
```

```
Tabla.m=rbind(c(33,28),c(31,25))
fisher.test(Tabla.m, alternative="two.sided")
```

```
##
##  Fisher's Exact Test for Count Data
##
## data:  Tabla.m
## p-value = 1
## alternative hypothesis: true odds ratio is not equal
to 1
## 95 percent confidence interval:
##  0.4301697 2.0977109
## sample estimates:
## odds ratio
##  0.9509033
```

En el caso que prefieran reportar las comparaciones entre los grupos como un test de homogeneidad \(\chi^2\), el resultado es el de la Figura 1:

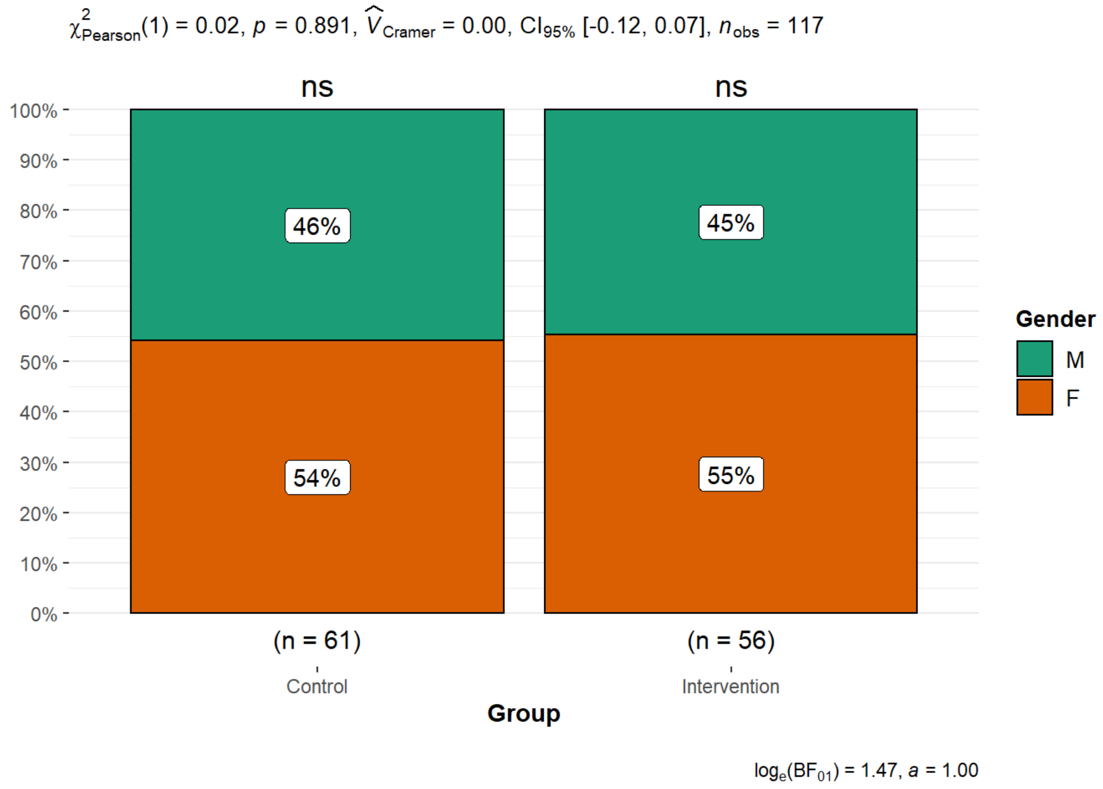

Figura 1: Distribución de la variable Género en los grupos: Realizamos un test \(\chi^2\) para analizar la homogeneidad en la distribución del Género entre los dos grupos y, como pueden ver en la leyenda, tanto la estimación puntual del estadístico como el intervalo de confianza, indican que no se puede rechazar la hipótesis nula de que el grupo Control y el grupo Intervención son comparables en lo que se refiere a la distribución del Género (p-valor = 0.891).

Comparación por edades:

En cuanto a la comparación de las edades entre los dos grupos, los resultados son los que se indican en la Figura 2. Para la evaluación de la normalidad en la distribución de las edades en ambos grupos, el test usado es el de Kolmogorov-Smirnov (con la corrección

Lilliefors) que está implementado en la librería nortest de R y que se aplica mediante la función lillie.test(). Para cualquier referencia adicional ver: https://cran.r-project.org/web/packages/nortest/nortest.pdf. Los resultados del test de normalidad para la distribución de edades en los grupos control e intervención son:

```
library(nortest)
lillie.test(datos[datos$Group=="Control",]$Age)
```

```
##
##  Lilliefors (Kolmogorov-Smirnov) normality test
##
## data:  datos[datos$Group == "Control", ]$Age
## D = 0.20277, p-value = 1.36e-06
```

```
lillie.test(datos[datos$Group=="Intervention",]$Age)
```

```
##
##  Lilliefors (Kolmogorov-Smirnov) normality test
##
## data:  datos[datos$Group == "Intervention", ]$Age
## D = 0.2311, p-value = 5.767e-08
```

Como pueden ver, ambos p-valores son menores a 0.05, por lo que la hipótesis nula (los datos se distribuyen normalmente) debe ser rechazada.

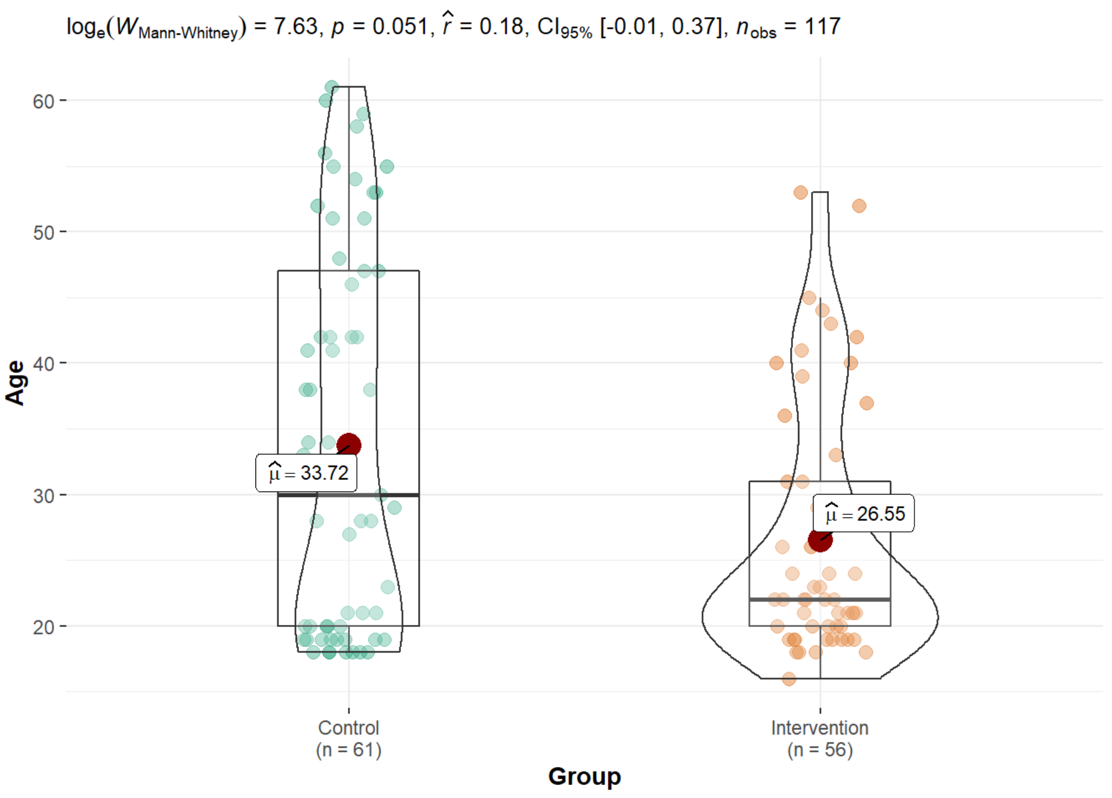

Figura 2: Dado las edades no están distribuidas normalmente en los grupos (p-valores<0.05 en el test Kolmogorov-Smirnov), se realizó una comparación de medianas usando el test de Mann-Whitney. Como pueden ver a partir de la estimación puntual del estadístico de la prueba (0.18) y del intervalo de confianza, no se observaron diferencias significativas en las medianas de las edades de ambos grupos (p-valor=0.051).

Comparación de la sensibilidad al olor en el test inicial.

Lo primero es hacer el test de normalidad para la sensibilidad medida antes de la prueba:

```
# library(nortest)
table(datos$Group,datos$Pretest)
```

```
##
##                  4  9 10 20 30
##   Control        0  0 60  1  0
##   Intervention  1  1 50  3  1
```

```
new.data=read.table(file = "new.data.txt", header = T)
table(new.data$Group,new.data$Sensitivity_test)
```

```
##
##                  4  5 10 15 20 28 30
##   Control        0  1 26  1  2  1  0
##   Intervention  1  0 26  0  2  0  1
```

```
lillie.test(datos[datos$Group=="Control",]$Pretest)
```

```
##
##  Lilliefors (Kolmogorov-Smirnov) normality test
##
## data:  datos[datos$Group == "Control", ]$Pretest
## D = 0.53455, p-value < 2.2e-16
```

De nuevo, los p-valores indican que los datos no son normales y la comparación de medianas debe hacerse usando el test de Mann-Withney:

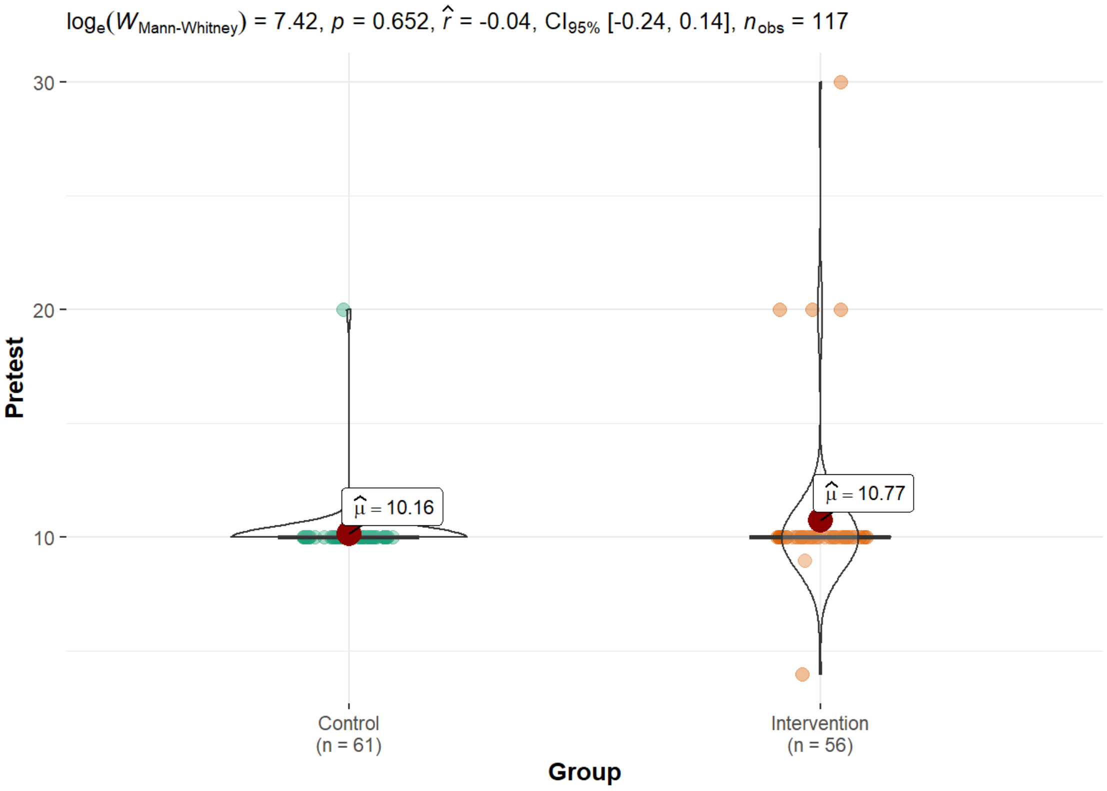

Figura 3: Los números de activaciones en la prueba inicial de sensibilidad no están distribuidos normalmente en los grupos (p-valores<0.05 en el test Kolmogorov-Smirnov), por lo que se realizó una comparación de medianas usando el test de Mann-Whitney. Al igual que en el caso de las edades, no se observaron diferencias significativas en las medianas del número de activaciones de ambos grupos (p-valor=0.652).

Análisis de la prueba

Una vez aclarado que los dos grupos son comparables, hay que entonces que comparar la distribución de APPROVED (los que no sintieron olor) con la de los FAILED (los que sintieron olor). De nuevo, esto se puede hacer comparando las proporciones entre los grupos control e intervención (usando el test exacto de Fisher), o con un test

χ^2.

En el primer caso hay que tomar en cuenta que:

```
table(datos$Group,datos$Outcome)
##
##                Approved Failed
##   Control             0     61
##   Intervention       53      3
prop.table(table(datos$Group,datos$Outcome), margin =
1)
##
##                  Approved     Failed
##   Control      0.00000000 1.00000000
##   Intervention 0.94642857 0.05357143
```

es decir, que de los $n_i=56$ voluntarios que hicieron la prueba con el dispositivo, hubo 53 que no sintieron el olor (un 95%), mientras que de los $n_c=61$ voluntarios que hicieron la prueba sin el dispositivo, todos lo sintieron.

En este caso, el test de comparación de proporciones (test exacto de Fisher) da como resultado $p-valor<2.2\cdot 10^{-16}, CI_{95\%}=(203,\infty)$ (recuerden que en el test de Fisher no se puede calcular el CI para la proporción sino para el Odds ratio)

Detalles:

```
Tabla.h=rbind(c(53,0),c(3,61))
fisher.test(Tabla.h, alternative="greater")
##
##  Fisher's Exact Test for Count Data
##
## data:  Tabla.h
## p-value < 2.2e-16
## alternative hypothesis: true odds ratio is greater
than 1
## 95 percent confidence interval:
```

```
##  203.0223      Inf
## sample estimates:
## odds ratio
##        Inf
```

Finalmente, si prefieren hacer el análisis con un test de homogeneidad:

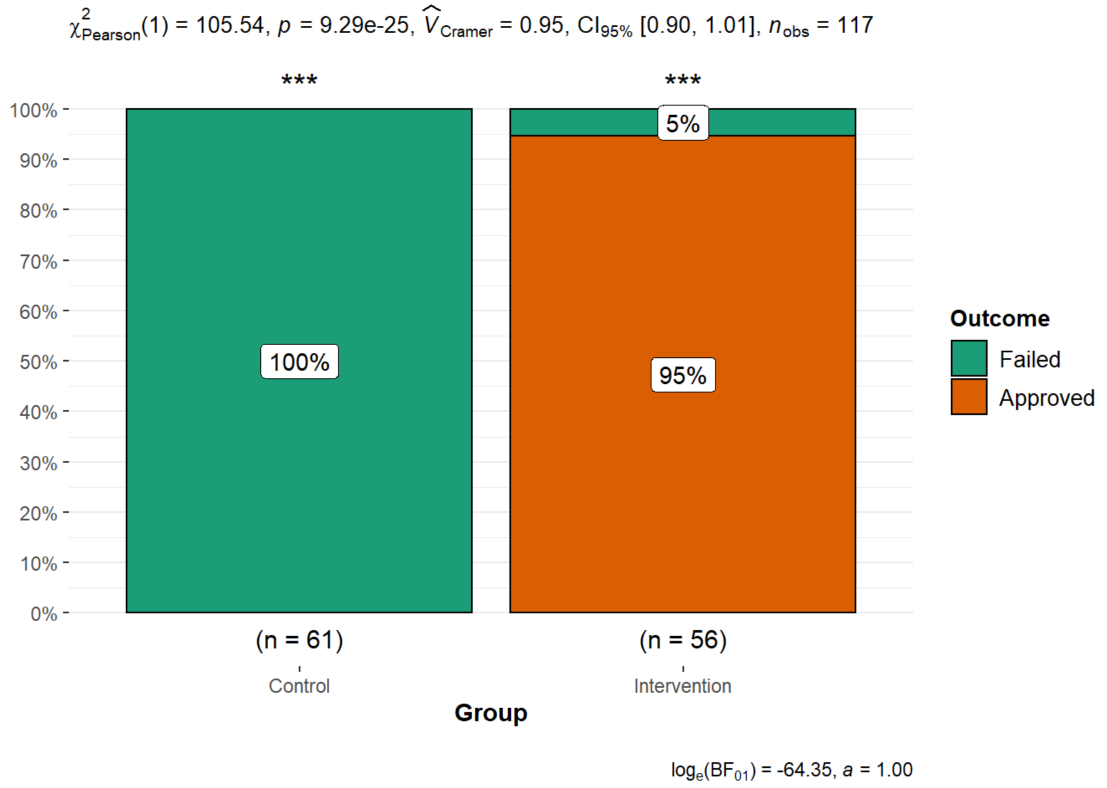

Figura 4: Distribución del resultado de la prueba en los grupos: Realizamos un test \(\chi^2\) para analizar la homogeneidad en la distribución de la respuesta entre los dos grupos y resulta un coeficiente de Cramer=0.95 (\(CI_{95\%}=(0.85,1.02)\)), con lo que se debe rechazar la hipótesis nula de que no hay diferencias en la distribución del resultado de la prueba entre el grupo Control y el

grupo Intervención (p-valor = $9.3\cdot 10^{-25}$).

La potencia del contraste, para un nivel de significancia 0.05, vuelve a ser del 100%:

```
library(pwr)
ES.h(0.95,0)
## [1] 2.690566
pwr.2p2n.test(h=2.606,n1=61, n2=56,
sig.level=0.05,power=NULL,alternative="greater")$power
## [1] 1
```
